# Stormwater Strategies: Cities Prepare Aging Infrastructure for Climate Change

**DOI:** 10.1289/ehp.119-a514

**Published:** 2011-12-01

**Authors:** Rebecca Kessler

**Affiliations:** Rebecca Kessler, based in Providence, RI, writes about science and the environment for various publications. She is a member of the National Association of Science Writers and the Society of Environmental Journalists.


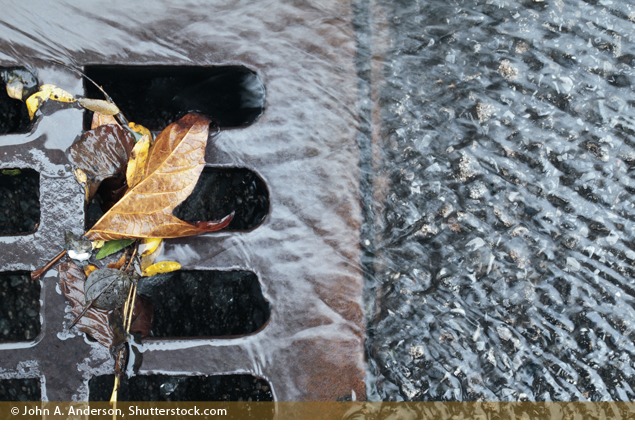
If it weren’t for the cement pipe segments nearly nine feet in diameter, oversize yellow backhoes, towering piles of sand, and yawning trenches scattering the streets, the section of Toronto near the junction of Connaught and Fargo avenues would look like an ordinary residential neighborhood. In spring 2011 this area of comfortable-looking contemporary houses surrounded by tidy landscaping was a construction zone as its storm sewers, most fewer than 20 years old, were ripped up and replaced with much larger pipes. The purpose of all the disruption? To prevent the flooding of basements and garages that has plagued these homes during unexpectedly heavy rains in recent years.

The area is just one of 32 across Toronto slated for storm sewer upgrades with a stringent new design standard. Whereas the old pipes were meant to capture the volume of water rushing off roofs, driveways, and streets during the kind of storm that would occur on average every 2–5 years, the new ones are tailored to so-called 100-year storms.^1^ These used to have a probability of occurring just once every century but now seem to be more frequent, according to Michael D’Andrea, director of water infrastructure management for the City of Toronto.

“That’s an extreme design standard, no matter how you look at it,” D’Andrea says, and shoehorning the necessary infrastructure into a densely developed city like Toronto is technically challenging and expensive—especially considering that the old pipes were still well within their useful life spans. “We’re rebuilding systems in an area of the city that, all things being equal, we shouldn’t have had to worry about for several decades to come,” he says.

**Figure:**
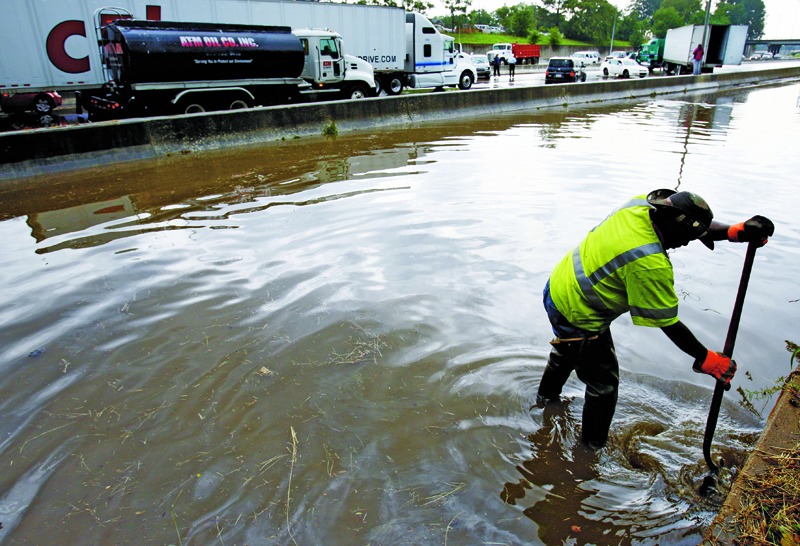
Memphis, Tennessee, 13 June 2011: A state Department of Transportation employee works to unclog a drain on the Madison Avenue ramp onto I-240, where the flow of traffic stopped due to high water covering the road. Heavy rain and wind had pounded Memphis earlier in the day, knocking out power to tens of thousands of residents and causing numerous traffic accidents (including the one in the background). The storm came just weeks after historic flooding of the Mississippi River overwhelmed the city’s sewer system, sending raw sewage into yards, streets, and the river itself.^33^ © Mike Brown/The Commercial Appeal/ZUMAPRESS.com

But extreme weather calls for extreme plans. City officials deemed the new standard necessary after two huge soakers, one in May 2000 and an even bigger one in August 2005, lit up the city’s switchboards with thousands of complaints of flooding and raw sewage backing up into basements. In fact, no fewer than eight so-called extreme weather events, with rainfall exceeding that of 25-year storms,^2^ have hit Toronto in the quarter-century since 1986. The 2005 downpour, which unleashed 6 inches of rain in about 3 hours, not only ruined basements but also damaged cars, broke water mains, washed away sections of road, flooded a wastewater treatment plant, and destroyed a sanitary sewer line, sending raw sewage into a creek.^3^ The Institute for Catastrophic Loss Reduction, a Toronto-based insurance-industry research institute, described it as the most expensive natural disaster ever to befall Ontario and the second most expensive in Canada, with damages exceeding Can$500 million.

“There’s no denying that in this particular area we’re seeing these extreme storms more frequently than we have in the past,” D’Andrea says. “It’s basically a call into action.”

Climate change is already affecting water utilities, according to a 2009 report from the National Association of Clean Water Agencies and the Association of Metropolitan Water Agencies, industry advocacy groups based in Washington, DC.^4^ A seemingly contradictory witches’ brew of more frequent and extreme storms, drought, and sea-level rise is beginning to stress some cities’ water infrastructure.

Although managers typically think first of the effects on drinking water supplies, many are realizing that their wastewater systems (the focus of this article) also will be keenly affected, with profound potential consequences for public health. In many places, these systems are already under strain from population growth, development, underfunding, and maintenance backlogs.^5^ At a time when North American cities are just beginning to assess what altered long-term weather patterns may bring, a handful, such as Toronto, have already committed to upgrading their wastewater systems with climate change in mind.

## Preparing for Change

Hundreds of cities are already addressing climate change by reducing their emissions of greenhouse gases.^6^ Lately, however, many have shifted into assessing their vulnerabilities to changing weather patterns, and some are starting to think about how to adapt, says Brian Holland, director of climate programs at ICLEI–Local Governments for Sustainability USA, a Boston-based nonprofit that offers technical assistance in climate change planning. Holland and a Canadian colleague estimate that perhaps 90 cities across the United States and Canada may be formally engaged in crafting adaptation plans.

This kind of planning is no easy task, and the stakes are particularly high when it comes to expensive water infrastructure. In a 2009 report, the U.S. Global Change Research Program predicted that over the coming decades many coastal areas will experience rising sea levels and increasing storm surges and that by and large the West and Southwest will grow drier, the Northeast and Midwest will grow wetter, and the entire nation can expect more severe storms.^7^ Yet while scientists have grown confident in making predictions over large areas and time frames, “downscaled” research about what to expect on the local level or more precisely when changes might unfold is scarce. That’s critical information for any planning process, says David Behar, climate program director for the San Francisco Public Utilities Commission and spokesman for the Water Utility Climate Alliance, a consortium of 10 large U.S. water providers. Because of the uncertainty, Behar says, most water utilities are still “living in an era of assessment rather than an era of adaptation.”

Changing conditions could affect waste-water systems in a variety of ways. Sea-level rise is one obvious concern in coastal areas, with waters having climbed up most U.S. shorelines by as much as 8 inches since 1958 and even more in a few places.^7^ High waters and storm surges may flood or damage coastal treatment plants or submerge outfall pipes, inhibiting discharge and causing backups into basements and streets. Perhaps the chief concern, however, is that heavy rains have become more frequent and intense across the entire United States—and the trend is only predicted to increase. Between 1958 and 2007 the number of days with very heavy precipitation^8^ increased by 8% in Hawaii on the low end and by 58% in the Northeast on the high end.^7^ Because of the way that many water systems are designed, rainfall has a big impact on water pollution and consequently on public health.

Drinking water supply systems are completely separate from storm and sanitary sewer systems. Newer cities often also have separate storm and sanitary sewer systems. These usually route raw residential and industrial sewage to treatment plants, while stormwater is funneled, untreated, into local waterways. However, some 772 U.S. cities—particularly older ones in the Northeast, Midwest, and Pacific Northwest—have combined storm and sanitary sewer systems.^9^ Ordinarily in these cities, all the wastewater is treated before discharge, but heavy rains can overwhelm the system, sending a cocktail of pathogens, active pharmaceutical ingredients, household chemicals, oil, pesticides, excess nutrients, and other pollutants straight into local receiving waters—a situation known as a combined sewer overflow (CSO).

CSOs are regulated and permitted by the U.S. Environmental Protection Agency (EPA) through the National Pollutant Discharge Elimination System, which requires municipal permittees to design and implement long-term CSO-control programs with the goal of gradually coming into compliance with the federal Clean Water Act. The increased heavy rainfall predicted for many parts of the continent is likely to make it harder for cities to meet their CSO reduction goals.^10^

Sandra McLellan, an associate scientist with the University of Wisconsin–Milwaukee School of Freshwater Sciences, has quantified the potential impact for Milwaukee. She and colleagues conducted a modeling study predicting that heavier rainstorms forecast for the region during the mid-twenty-first century could unleash up to 20% more untreated wastewater via CSOs.^11^

Failing wastewater infrastructure poses another problem: The EPA predicts that the percentage of U.S. wastewater pipe that will be in “poor,” “very poor,” or “life elapsed” (older than its predicted life span) condition will rise from 23% in 2000 to 45% in 2020.^12^ As of 2008, the agency calculated that repairing the nation’s sanitary and stormwater sewer infrastructure would cost $298 billion.^13^ Climate change could raise the tab even higher. In their 2009 report, the National Association of Clean Water Agencies and the Association of Metropolitan Water Agencies estimated that adapting to climate change through 2050 could cost U.S. water utilities between $448 billion and $944 billion—and that doesn’t account for responses to weather emergencies.^4^

McLellan and colleagues have demonstrated one unexpected way that failing infrastructure is already causing pollution problems. In an article published in August 2011 the team reported finding very high human fecal pathogen levels in all 45 stormwater outfalls they sampled in an area of Milwaukee with a separate sewer system.^14^ The implication is that sewage is leaking out of compromised underground sanitary sewer pipes and into nearby stormwater pipes, according to the authors. In July 2011 researchers from the University of California, Santa Barbara, reported a similar effect during dry weather after adding dye to sanitary sewers. The dye turned up in nearby storm drains, which subsequently tested positive for human fecal contamination.^15^ Those findings show that increased rainfall could pose a pollution problem even for cities with separated sewer systems, which often don’t treat stormwater before releasing it, McLellan says.

The bottom line, according to McLellan, is that, between CSOs and failing infrastructure, climate change will not be good for water quality. “If we see on average more intense rainfalls or more extreme rainfalls or a higher number of rainfalls, we’re going to have more pathogens entering our waterways,” she says.

People can be exposed to waterborne pathogens recreationally by swimming, beachgoing, boating, and other activities. Drinking water supplies can also be contaminated. For instance, an outbreak of some 1,450 cases of gasteroenteritis occurred on Ohio’s South Bass Island in Lake Erie during the summer of 2004. Judging by the menagerie of human pathogens detected, groundwater drinking supplies had become contaminated with sewage from local wastewater treatment plants and septic tanks after record-high rains earlier in the spring.^16^ Such outbreaks, along with major natural disasters including the catastrophic 2010 floods in Pakistan that left millions of people stricken with flood-related disease, are a reminder of the potent connections between climate, water, and human health. (The floods ravaging Thailand at press time had not yet caused any reported disease outbreaks, although with a significant portion of the country inundated as of mid-November 2011,^17^ there remains a distinct threat of waterborne disease.)

**Figure:**
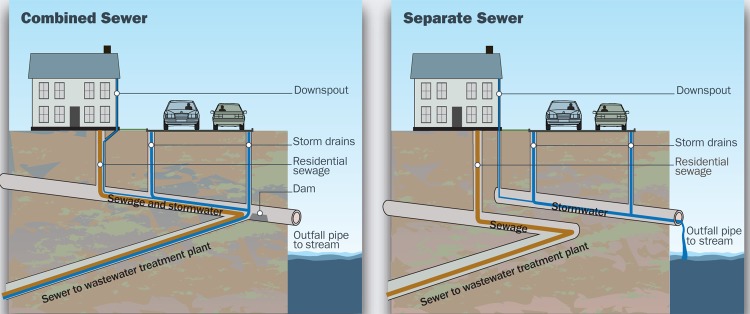
Combined sewer systems collect rainwater and sewage in the same pipe. The mixed waste is transported to a plant where it is treated before being discharged. If the volume of wastewater in a combined sewer system exceeds capacity, as during a heavy rainstorm, the system is designed to discharge the excess—which can include raw sewage and toxic chemicals—directly into nearby water bodies. In a separate sewer system, sewage is directed to a treatment plant via one pipe while untreated rainwater is discharged into water bodies via another. Even in these systems, however, raw sewage may make its way into water ways, perhaps by migrating from compromised pipes. Joseph Tart/EHP

A sizeable body of research, including some by McLellan, has shown a clear association between heavy rainfall and disease outbreaks.^18^ For instance, a 2001 analysis of 548 waterborne-disease outbreaks reported in the United States between 1948 and 1994 showed that 68% were preceded by rainfall events above the 80th percentile.^19^ Similarly, a 2006 article reported that extreme rain events more than doubled the risk of waterborne disease outbreaks in Canada between 1975 and 2001.^20^ McLellan hopes to take this line of research a step further by contributing to a Wisconsin-wide risk assessment, now under way. The project is intended to predict rates of waterborne illness from rainfall changes projected under climate change scenarios through the middle and end of this century, as well as to determine what level of precipitation is likely to have serious public health consequences.

## Cities Go Green

Toronto’s effort to prevent basement flooding during extreme rains is just one aspect of an ambitious 25-year plan focused on complying with provincial law to control CSOs, cleaning up the city’s waterways and beaches, and shaking off its unwelcome designation as an Area of Concern under the Canada–United States Great Lakes Water Quality Agreement, a title it has held since 1987.^21^ With a storm sewer system that totals some 2,800 miles and drains to 2,600 outfalls, this is a serious undertaking. And with about one-fifth of the city’s water infrastructure dating to the 1930s or earlier, it comes on top of a maintenance backlog of Can$1.7 billion.

The plan, called the Wet Weather Flow Master Plan, originated in 2003 with a billion-dollar price tag, but in August 2011 the Toronto city council approved its next phase at nearly Can$3.5 billion. The plan includes many “green infrastructure” approaches that mimic natural processes, letting stormwater evaporate or percolate into the ground and avoid the wastewater system altogether.^21^ These include disconnecting buildings’ downspouts, planting trees, and installing bioswales and green roofs.^22^ Restoring a total of about 1 kilometer of streams per year, replanting and reshaping their banks and flow patterns to combat property-threatening erosion and improve wildlife habitat also is part of the program.^21^

The plan also includes major “grey infrastructure” projects—that is, engineered facilities and structures. The biggest of these will duplicate a major, aging sanitary sewer artery to serve as a backup and provide additional stormwater capacity, as well as install tunnels, underground storage tanks, and a new treatment facility. This huge venture, which involves more than 14 miles of new tunnel, should virtually eliminate CSOs to the city’s beautiful central waterfront area and its stretch of the Don River—now among the most degraded rivers in Canada, according to the Toronto and Region Conservation Authority.^23^ And finally there are projects that fall somewhere in between green and grey, including constructed ponds and wetlands for stormwater storage and filtration.^24^

Toronto’s efforts are already paying off. Eight of its 11 beaches have made a remarkable turnaround, earning “blue flag” status from the Canadian nonprofit Environ-mental Defence for meeting strict water quality standards.^25^ And the average percentage of days on which beaches were cited as unsafe for swimming due to high fecal coliform counts declined from 49 in 2000 to 21 in 2010, according to Mahesh Patel, a manager with Toronto’s public health department. Back in 2003, when Toronto first unrolled its stormwater plan, improving water quality was the main concern. But the city has since come to regard the plan as a critical component of its climate change adaptation strategy, adopted in 2007.^26^

Other North American cities are on the same path, although only a handful have gone so far as to specifically account for climate change conditions in project designs. The Massachusetts Water Resources Authority, for instance, got into the game early with Boston’s immense Deer Island Sewage Treatment Plant, completed in 2001. In designing the plant, engineers elevated it an additional 1.9 feet to accommodate sea-level rise predicted by 2050, the end of the facility’s planned life span.^7^ In San Francisco, where sea level has already risen 8 inches over the past century, high tides have occasionally started inundating wastewater outfall pipes that empty into the ocean, sending saltwater into the treatment system, according to Behar, of the San Francisco Public Utilities Commission. He says the problem is expected to worsen, so the city decided to retrofit some outfalls with backflow-prevention devices at a cost of up to $40 million.

**Figure:**
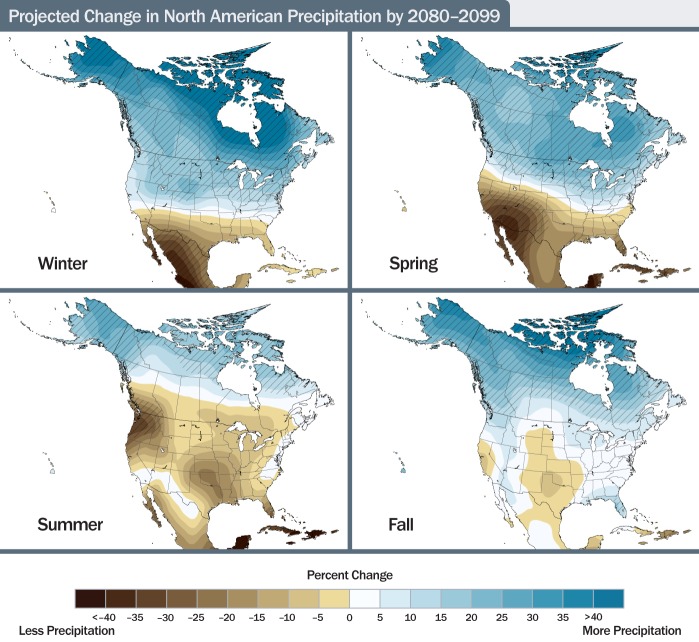
**Projected Change in North American Precipitation by 2080–2099** These maps show projected future changes in precipitation relative to the recent past as simulated by 15 climate models from the World Climate Research Programme Coupled Model Intercomparison Project 3. The simulations assume a higher emissions scenario. Hatched areas reflect seasons and locales where there is greater agreement among models that projected changes are likely. There is less confidence in exactly where the transition between wetter and drier areas will occur. Source: Karl et al. (2009)^7^

Rainy Seattle added 6% to the volume of planned immense underground CSO storage tanks to handle extra precipitation expected with climate change, according to Paul Fleming, manager of Seattle Public Utilities’ Climate and Sustainability Group. But with little certainty about exactly how much more rain will come, the city is also investing in a number of “no-regrets” meas-ures that will improve stormwater management no matter what, Fleming says. These include hiring a meteorologist and developing a real-time weather system called RainWatch^27^ to document and forecast rain accumulation so the city can send out crews to, for instance, check that storm drains are working or increase pumping in hard-hit zones.

Like many cities, Seattle is tackling its CSO problem on a variety of fronts to meet EPA water-quality mandates, and climate change has become one more compelling reason to do so, Fleming says. Numerous other cities have adopted that logic regardless of where they may be in the process of studying how climate change could affect them and what they might do to adapt. Among them are major metropolises such as New York, Los Angeles, and Chicago, and many are investing aggressively in green stormwater-control initiatives as a hedge against climatic uncertainty.

Philadelphia may be unique in its heavy emphasis on green infrastructure.^28^ The state of Pennsylvania approved the city’s 25-year Green City, Clean Waters plan in June 2011. Of the plan’s $2.4-billion budget, city officials allocated 70% to green infrastructure, compared with just 14% for treatment plant upgrades.

The city regards the plan not only as a solution to its formidable water-quality problems but also as a climate change adaptation measure, says Paula Conolly, a consultant at the Philadelphia Water Department. “Green stormwater infrastructure is certainly easier and more feasible to tweak to changing conditions than is a more traditional grey solution, like a tunnel. If our capacity needs to increase, if we need more infrastructure to handle increased frequency or intensity of storms, for example, then green stormwater infrastructure is the way to do that,” she explains. “Every dollar we’re spending there is a dollar spent toward climate change adaptation.”

Green stormwater initiatives can offer other benefits, too. Green roofs reduce energy use and keep cities cooler, and parks and tree-lined streets improve property values and air quality.^29^ And then there are the jobs. In October 2011 a report by the advocacy group Green For All, based in Oakland, California, and Washington, DC, calculated that investing $188 billion in green stormwater projects over the next 5 years would create nearly 1.9 million jobs, from arborists and installers of green roofs to sewer-pipe repairers, and generate about $265 billion in economic activity.^30^

When it comes to improving water quality, traditional grey wastewater infrastructure undoubtedly works, says Tiffany Ledesma Groll, a consultant to the Philadelphia Water Department on the Green City, Clean Waters plan. But with a green approach, she says, “You have all these [positive] impacts on society on a social, economic, and environmental level. . . . Why wouldn’t you want to do it this way?”

## Waste Not

As researchers, city officials, and utility managers grapple with an environment that is changing around them, a new way of thinking about sewage and stormwater is emerging. Wastewater is coming to be viewed as a resource and not just a polluting nuisance. After all, it’s loaded with potentially revenue-generating nutrients,^31^ energy,^32^ and, obviously, water, which in many parts of North America is already becoming scarce as climate change takes hold, says Joan B. Rose, a microbiologist who codirects the Center for Advancing Microbial Risk Assessment and the Center for Water Sciences at Michigan State University.

**Figure:**
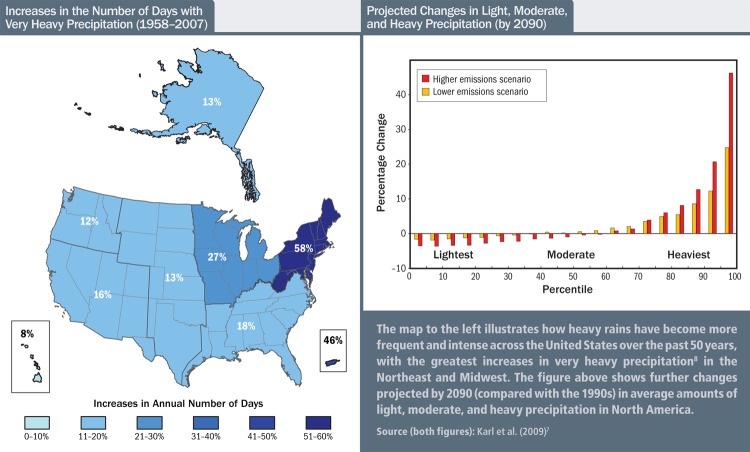
**Increases in the Number of Days with Very Heavy Precipitation (1958–2007) | Projected Changes in Light, Moderate, and Heavy Precipitation (by 2090)** The map to the left illustrates how heavy rains have become more frequent and intense across the United States over the past 50 years, with the greatest increases in very heavy precipitation^8^ in the Northeast and Midwest. The figure above shows further changes projected by 2090 (compared with the 1990s) in average amounts of light, moderate, and heavy precipitation in North America. Source (both figures): Karl et al. (2009)^7^

New technology is needed to harvest those resources efficiently as well as to eliminate such newly recognized forms of water pollution as active pharmaceutical ingredients and pathogens that can creep through treatment unscathed, says Rose, who coauthored the 2001 paper correlating disease outbreaks and heavy rainfall in the United States^19^ and studied the South Bass Island outbreak. “We have not put enough science and technology into wastewater as we’ve needed to,” she says, but there’s no time like the present to invest, even in a struggling post-recession economy.

“Germany actually led the revolution in wastewater treatment in the 1800s,” Rose says. “What if the United States led the new revolution in technology that the world needs? It’s just an incredible opportunity.”
